# Updated Meta-Analysis Assessing Effects of Baduanjin on Cardiopulmonary Functions of Patients with Coronary Heart Disease

**DOI:** 10.1155/2022/3913082

**Published:** 2022-09-29

**Authors:** Dongsheng Wang, Jun Xu

**Affiliations:** ^1^Physical Education Department, Weifang University, Weifang 261061, China; ^2^College of Sports and Art, Shandong Sport University, Jinan 250102, China

## Abstract

**Background:**

Baduanjin is a kind of moderate-intensity aerobic exercise, but its effect on the cardiac rehabilitation (CR) of patients with coronary heart disease (CHD) is controversial. Furthermore, the small sample size of a single study and the inconsistent selection of evaluation indicators among different studies all promote the necessity of conducting a meta-analysis.

**Objective:**

This meta-analysis aims to explore whether Baduanjin can effectively improve the cardiopulmonary function in patients with CHD and to assess the extrapolation of the results.

**Methods:**

Both English and Chinese databases were used for literature retrieval. The Cochrane Collaboration tool was used to evaluate the methodological quality of the included literature. A weighted mean difference and a 95% confidence interval were used to assess the effects of Baduanjin on cardiopulmonary function based on multiple CR indicators. Cochran's *Q* and *I*^2^ tests were used for the heterogeneity test. A funnel plot and an Egger test were used to evaluate publication bias.

**Results:**

After literature retrieval, a total of 12 literature papers were included in this meta-analysis, but their methodological quality was unsatisfactory. By comparing differences between CHD patients with and without Baduanjin, we found that levels of 6MWT, LVEF, NT-proBNP, VO_2_, peak VO_2_, MVV, and VE, AT were significantly improved in the experimental group, but heterogeneity exists among included studies. Results of subgroup analyses were consistent with combined estimates and suggested a significant effect of Baduanjin on LVEF. The Egger test indicated no significant publication bias.

**Conclusion:**

Baduanjin is beneficial to CR in CHD patients, but multiple region-based high-quality studies are necessary to verify the results.

## 1. Introduction

Coronary heart disease (CHD), also known as coronary artery disease, is the most common cause of death worldwide, affecting more than 11 million people in China, and the number of patients is increasing by 20% every year [1]. CHD is a risk factor for acute myocardial infarction, unstable angina, cardiac arrest, and heart failure [2]. Cardiac rehabilitation (CR) is complex and multifaceted support for patients with CHD to improve their health and prognosis status [3]. Of which, exercise training is one of the core modes of CR and has been proven to effectively enhance the patient's endurance and prolong their physical activity time [4]. Its potential benefits also include improved endothelial function, myocardial blood flow reserve, and psychological stress in patients with CHD [5]. A meta-analysis of 14,486 patients with CHD found that exercise-based CR can reduce cardiovascular mortality and the risk of hospitalization [6]. Bai and Wang concluded that lighter activity intensity was independently correlated with health indicators of cardiovascular metabolism and had the strongest correlation with triglycerides and lipid accumulation products [7]. However, the training intensity that CHD patients receiving exercise-based CR therapy can tolerate is an important concern, and an appropriate stimulus is needed to trigger positive physiological application in order to improve peak VO_2_ as much as possible [8]. Therefore, optimizing exercise prescription is significant for the recovery of cardiopulmonary function in patients with CHD.

Baduanjin, a traditional Chinese qigong, has a history of more than 1,000 years as a form of exercise. It is characterized by symmetrical body postures and movements and allows the interaction between thought and breath to coexist in a harmonious manner [9]. Baduanjin is a set of independent and complete fitness systems, and its eight decomposing movements have their own effects on specific parts of the body and, therefore, play an integrated adjustment [10]. According to the gold standard of exercise intensity VO_2_, Baduanjin is a kind of moderate-intensity aerobic exercise and also a safe and effective family CR exercise mode, with an average energy consumption of 23.3 ± 4.4 kcal [11]. The physical health benefits of Baduanjin include treating hyperlipidemia, ischemic stroke, sleep disorders, and knee osteoarthritis, as well as improving mental health, quality of life, balance, and flexibility of patients [10, 12]. In addition, studies also highlighted the physiological benefits of Baduanjin practice, including improvements in cardiopulmonary functions [13]. There have been related randomized controlled studies (RCTs) to explore the effect of Baduanjin on cardiopulmonary function recovery in patients with CHD, but the sample size of a single study is relatively small (ranging from 40 to 120), and the selection of evaluation indicators is inconsistent [14–16]. Meanwhile, it is still controversial whether CHD patients with percutaneous coronary intervention (PCI) had a significant difference in left ventricular ejection fraction (LVEF) with and without Baduanjin [15, 16]. Therefore, it is necessary to further expand the sample size by gathering samples of eligible RCTs as many as possible, to discuss the effect of Baduanjin on cardiopulmonary function recovery in patients with CHD.

This study summarized several RCT reports and conducted a more comprehensive and objective evaluation of the difference between Baduanjin on cardiopulmonary function indicators through this meta-analysis, so as to explore the impact of Baduanjin on cardiopulmonary function recovery for patients with CHD. Additionally, we also assessed the extrapolation of the results so as to promote the benefits of this sport globally. This study also provides evidence for patients with CHD to choose a superior pattern of rehabilitation nursing.

## 2. Methods

All procedures were performed in compliance with the Preferred Reporting Items for Systematic Reviews and Meta-Analyses guidelines [17].

### 2.1. Retrieval Strategies

Both English and Chinese databases, including PubMed, Embase, the Cochrane library, Wanfang data, China National Knowledge Infrastructure, China Science and technology journal database, and China Biology Medicine disc were used in this study for bibliographic retrieval. The search keywords included Baduanjin AND (“coronary heart disease” OR “ischemic heart disease” OR “Ischemic cardiomyopathy” OR “myocardial infarction” OR “acute coronary syndrome” OR stenocardia OR “coronary artery disease”). The retrieval procedures and results of the English databases are shown in Tables S1–S3. Search deadline is August 9, 2022, with no language restrictions. Furthermore, the paper literature and the references included in relevant reviews were manually searched in this study.

### 2.2. Literature Screening

Inclusion criteria: (1) the subjects were patients with CHD; (2) the studies discussed the difference of intervention effect on cardiopulmonary function in CHD patients of Baduanjin vs. control (maintain original habit of exercise), or between Baduanjin + exercise rehabilitation (ER, including aerobic training, aerobic walking training, low intensity aerobic combined resistance exercise, et al.) vs. ER; (3) randomized controlled trials; and (4) the studies reported one or more of the following outcomes: N-terminal pro-B-type natriuretic peptide (NT-proBNP), LVEF, 6 min walk test (6MWT), peak VO_2_, anaerobic threshold (AT) VO_2_, maximal voluntary ventilation (MVV), AT ventilation volume (VE, AT), O_2_ pulse, metabolite equivalents (METs), and AT METs.

Exclusion criteria: (1) reviews, conference abstracts, and comments; (2) the postintervention measurement results (mean ± standard deviation) were not reported or could not be obtained according to literature information; and (3) for repeated publications or multiple articles with the same data, only the one with the most complete research information was included and the rest were excluded.

### 2.3. Data Extraction and Quality Assessment

Two researchers independently conducted literature screening and data extraction according to the above screening criteria. Information to be extracted includes: the first author, publication year, country, demographic characteristics of study subjects (age, gender, sample size, disease course, and CHD types), intervention plan, follow-up period, and study outcomes. The Cochrane Collaboration's tool for assessing risk was utilized to evaluate the methodological quality of the included studies [18]. In the case of disagreement in the process of literature data extraction and quality evaluation, a consensus was reached after a group discussion with the third author.

### 2.4. Statistical Analysis

Weighted mean difference (WMD) and 95% confidence interval (95%CI) were used as the effect size indicators of continuous variables (including NT-proBNP, LVEF, 6MWT, and other cardiopulmonary function indicators) to evaluate whether the intervention effect of Baduanjin on the cardiopulmonary function of CHD patients was statistically significant. The heterogeneity test was then generated using Cochran's *Q* test and *I*^2^ test [19]. The meta-analysis was performed using a random effect model when a significant heterogeneity exists at *P* < 0.05 and/or *I*^2^ > 0.5. If heterogeneity was not significant (*P* ≥ 0.05 and *I*^2^ ≤ 0.5), the fixed effect model was used for meta-analysis [20]. The effects of follow-up time, intervention plan, and disease course on heterogeneity and combined outcomes were then assessed by subgroup analysis. Finally, the funnel plot and Egger test were used to evaluate whether there was significant publication bias among included studies [21]. Statistical analysis was generated using RevMan 5.3 and Stata12.0.

## 3. Results

### 3.1. Literature Retrieval

**Figure 1:**
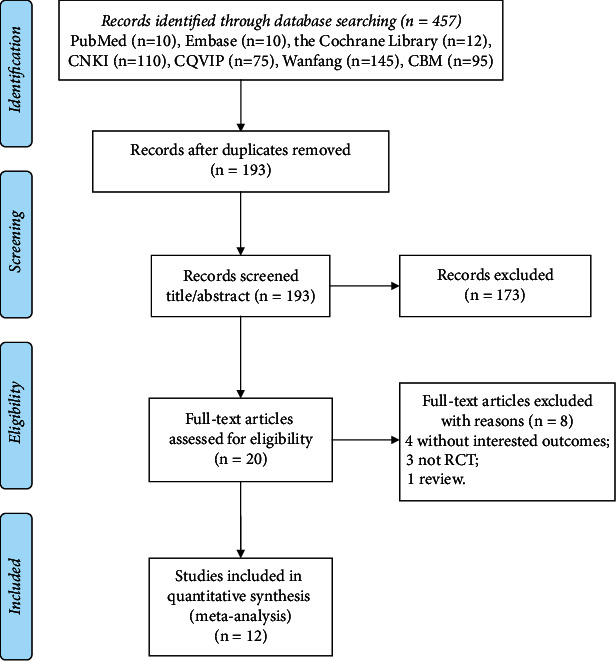
The literature search procedures and results of this meta-analysis.

The literature search process and results are described in Figure 1. Using the public databases, a total of 457 pieces of literature were searched, of which 193 duplicate articles were eliminated, and 173 studies did not meet inclusion criteria after browsing titles and abstracts. Then, eight of the remaining 20 papers were eliminated after the full text reading. Among them, four studies did not report study outcomes; three studies were not RCTs; and one was a review. Finally, a total of 12 pieces of literature [14–16, 22–30] were included in this meta-analysis.

### 3.2. Study Characteristics and Quality Assessment

As shown in Table 1, the 12 included studies were all carried out in China and published between 2016 and 2021 with a sample size of 40–120. By gathering these studies, a total of 468 cases and 460 controls were included in this meta-analysis. Among them, Wang [26] reported the number of male and female patients in all subjects, while other studies reported the number of male and female patients in the experimental group and control group, respectively. In terms of gender, there were 588 male patients and 340 female patients in all enrolled studies. In terms of CHD types, the research objects of five literature [15, 16, 23–25] were CHD with PCI; two [14, 29] were CHD with chronic heart failure (CHF); one [27] was stable CHD with PCI or coronary artery bypass grafting (CABG), one [30] was CHD with percutaneous transluminal coronary angioplasty (PTCA); and the remaining three studies [22, 26, 28] did not report the specific CHD types. In addition to routine nursing (including diet guidance, pharmacological nursing, and mental nursing), three studies [14, 24, 28] reported the effects of Baduanjin on cardiopulmonary function in CHD patients of Baduanjin + ER vs. ER (AWT [14], AT [28], and low-intensity aerobic combined resistance exercise [24]), while the rest of the studies explored the differences between patients with and without (maintain the original habit of exercise) Baduanjin. The follow-up time of RCTs was 0.5 to 6 months. The detailed information on age, disease course, exercise rehabilitation program and the New York Heart Association (NYHA) classification are shown in Table 1.

The methodological quality evaluation results of the included literature are shown in Figure S1. Except for the study of Chen et al., other studies have relatively high degrees of bias. As the included studies did not clearly describe whether allocation concealment and outcome measurement blindness were implemented and did not measure the blinding of participants and personnel, the bias therefore mainly focused on selection bias, performance bias, and detection bias. Overall, the methodological quality of the included studies was low.

### 3.3. Meta-Analysis on Effects of Baduanjin on Outcome Indicators for Patients with CHD

**Figure 2:**
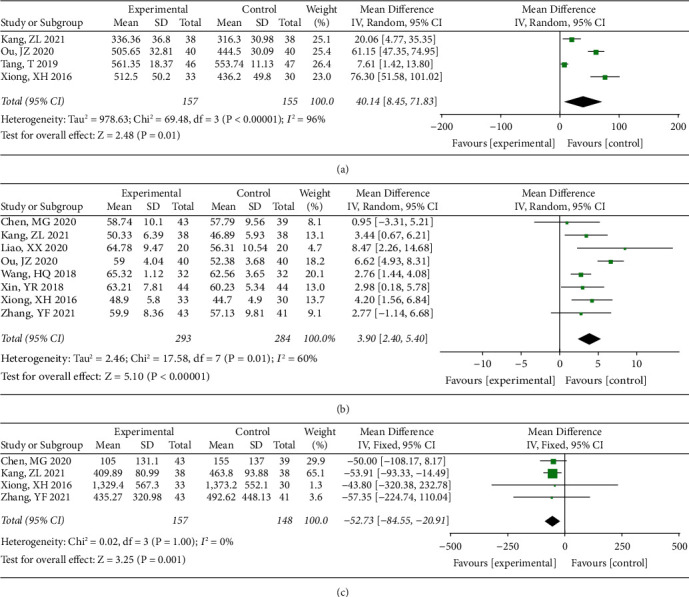
(a) The forest plots showed differences in 6MWT, (b) LVEF, and (c) NT-proBNP between CHD patients with and without Baduanjin.

The differences in 6MWT, LVEF, and NT-proBNP were then compared between CHD patients with and without Baduanjin. Four studies [14, 23, 25, 29] reported the difference in 6MWT, and *I*^2^ = 96% and *P* < 0.00001 indicated a significant heterogeneity among these studies. Therefore, the random effect model was conducted and the combined results suggested WMD (95% CI) = 40.14 (8.45, 71.83) meters and *P* = 0.01 (Figure 2(a)). Eight studies [14–16, 23, 26, 28–30] reported the differences in LVEF, but the significant heterogeneity existed (*I*^2^ = 60%, *P* = 0.01), and the random effect model suggested the combined results of WMD (95%CI) = 3.90 (2.40, 5.40)% and *P* < 0.00001 (Figure 2(b)). Four studies [14, 15, 29, 30] reported the differences in NT-proBNP, and there was no significant heterogeneity among these studies (*I*^2^ = 0%, *P* = 1.00), followed by the combined results of WMD (95%CI) = −52.73 (−84.55, −20.91) pg/mL and *P* = 0.001 using the fixed effect model (Figure 2(c)). These results indicate that Baduanjin may affect the levels of 6MWT, LVEF, and NT-proBNP in patients with CHD, despite some heterogeneities.

**Figure 3:**
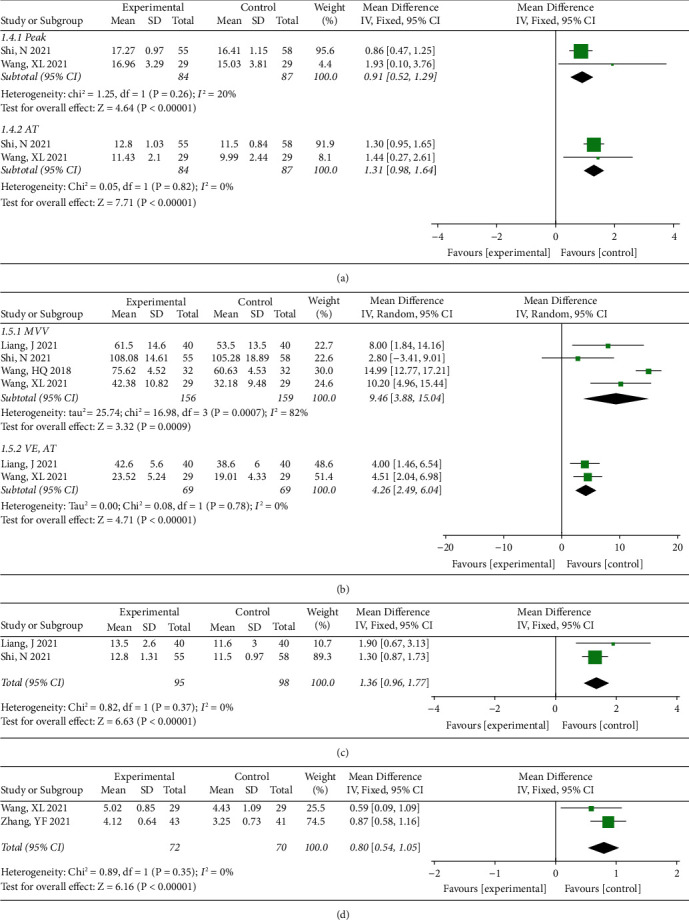
Effects of Baduanjin on indicators including peak VO_2_ (a), AT VO2 (a), MVV (b), VE, AT (b), O_2_ pulse (c), and METs (d) of patients with CHD.

Then two studies [24, 27] were included to compare the difference in VO_2_, while peak VO_2_ and AT VO_2_ showed no significant heterogeneity (*I*^2^ < 50%, *P* > 0.05) with the combined estimate of WMD (95%CI) = 0.91 (0.52, 1.29) ml/kg/min and WMD (95%CI) = 1.31(0.98, 1.64) ml/kg/min, respectively, and *P* < 0.00001 (Figure 3(a)). By comparing the difference in MVV (Figure 3(b)), the combined results of four studies [22, 24, 26, 27] were WMD (95%CI) = 9.46 (3.88, 15.04) L/min, *P*=0.0009 with a significant heterogeneity (*I*^2^ = 82%, *P*=0.0007). Figure 3(b) also shows the differences in VE, AT based on two studies [22, 27] and the combined estimate were WMD (95%CI) = 4.28 (2.49, 6.04) L/min, *P* < 0.00001 with a nonsignificant heterogeneity (*I*^2^ = 0%, *P*=0.78). To summarize the differences in O_2_ pulse [22, 24] (Figure 3(c), and METs [27, 30] (Figure 3(d)), it was found that studies were not significantly heterogeneous (I^2^ < 50%, *P* > 0.05), and the combined results were WMD (95%CI) = 1.36 (0.96, 1.77) mL/time, *P* < 0.00001, and WMD (95%CI) = 0.80 (0.54, 1.05), *P* < 0.00001 for O_2_ pulse and METs, respectively. The differences in these indicators between CHD with and without Baduanjin indicated that Baduanjin can effectively improve the recovery of cardiopulmonary function in CHD patients.

### 3.4. Subgroup Analysis

Because of the limited literature on most outcome indicators, the subgroup analysis was only generated on LVEF in this meta-analysis. As shown in Table 2 and Figure S2, it was found that follow-up time, rehabilitation scheme, and new-onset status are not the sources of the significant heterogeneity. Furthermore, in the subgroups of follow-up time <1 month and new-onset, the estimates were not significant (*P*=0.71), but the results in other subgroups were statistically significant (*P* < 0.05), consistent with the original combined results.

### 3.5. Publication Bias Test

**Figure 4:**
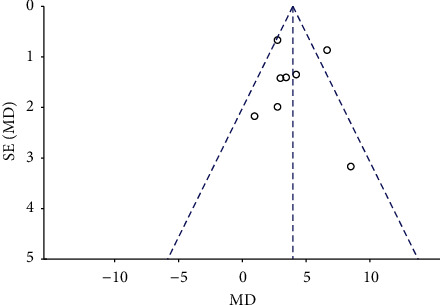
The funnel plot shows the publication bias on the outcome indicators of LVEF.

Publication bias was analyzed on LVEF, the most studied outcome index, using the funnel plot and Egger test. The results show the symmetric scatter distribution in the funnel plot with a *P*=0.983 > 0.05 of the Egger test (Figure 4). Qualitative and quantitative tests indicated that there was no significant publication bias on LVEF among the included studies.

## 4. Discussion

With the improvement of living standards, CHD has increasingly become an important threat to humans all over the world, and exercise-based CR has become an effective postoperative treatment for patients with CHD. It has been proven that regular physical activity can improve athletic ability as well as increase peak oxygen consumption (+15%) and peak anaerobic threshold (+11%) [31–33]. In addition, peak VO_2_ was associated with lower mortality in patients with coronary artery disease, and the survival rate of CHD patients can increase by nearly 15% with each increase of 1 mL/kg/min of exercise capacity [34, 35]. As a highly adaptable form of exercise, Baduanjin can be carried out anywhere and at any time, without special equipment or a large time investment. Therefore, it is beneficial to incorporate Baduanjin into a comprehensive CR program, but its effect on postoperative cardiac recovery in patients with CHD is worth discussing.

Through this meta-analysis, we found significant differences in the levels of peak VO_2_, LVEF, 6MWD, and other rehabilitation indicators between CHD patients who received and did not receive Baduanjin training, suggesting that Baduanjin can effectively promote the recovery of cardiopulmonary function in CHD patients. Wang et al. supported our conclusion and suggested that Baduanjin exercise can improve LVEF, cardiac output, and stroke output, and reduce resting myocardial oxygen consumption in elderly patients [36]. We also performed a subgroup analysis of LVEF, and the results were consistent with the combined estimate. However, in terms of the 6MWD indicator, the current research conclusions are still controversial. Zhang and Chang suggested no statistically significant difference in the level of 6MWD between exercise and nonexercise CHD patients with PCI (*P* = 0.71), and it is therefore uncertain whether exercise can improve cardiac function or reduce the incidence of adverse cardiovascular events in patients with CHD after PCI [37]. In addition, the cardiopulmonary exercise test is a practical tool to evaluate the exercise capacity of cardiac failure patients, in which peak VO_2_, VO_2_, VAT, and VE/VCO_2_ are important indicators [38]. Yu et al. enrolled 120 patients with ischemic heart failure in CR training and found that patients who received an additional 45 minutes of Baduanjin training showed significant improvement in exercise ability indexes, including 6MWT, peak VO_2_, VE/VCO_2_, LVEF, and NT-proBNP [39]. This conclusion demonstrates the benefit of Baduanjin in improving cardiopulmonary function in patients with heart failure, which is considered to be an end-stage state of cardiovascular disease, including CHD [40], thus supporting our findings to a certain degree.

To highlight the advantages of this study, we resolved the dispute over the inconsistent results of the original studies with objective results. In this meta-analysis, the combined results of all outcome indicators suggested that Baduanjin can significantly improve cardiopulmonary functions in CHD patients. The results of subgroup analysis showed that for CHD patients, Baduanjin exercise with or without routine rehabilitation training had a significantly better recovery effect on LVEF than the control group. Furthermore, there was no significant publication bias in this study, and the results were highly reliable. However, there are still some defects in this study. First of all, there was relatively large heterogeneity among the included studies, and differences in follow-up time, CHD type, and rehabilitation plan would affect the results of this meta-analysis. Second, the methodological quality of the included studies was unsatisfactory, and the control of selection bias, implementation bias, and measurement bias was not carried out strictly according to the standard of RCT. Additionally, the sample size and rehabilitation indicators except for LVEF of included studies are limited, which were not suitable for auxiliary analyses such as subgroup analysis and publication bias test. Therefore, the credibility and stability of meta-analysis results need further exploration and verification. Finally, all the included studies were carried out in China. The main reason is that Baduanjin is a traditional health exercise in China, and it is not as popular as Tai Chi. Therefore, there are few available data based on other regions and countries, which leads to a poor extrapolation of the results. High-quality RCTs in other regions and with larger sizes are considerable in further research, to evaluate whether Baduanjin has a similar effect on the cardiopulmonary function of CHD patients from other ethnic groups.

## 5. Conclusion

This meta-analysis indicated that the exercise of Baduanjin is beneficial in improving the recovery of cardiopulmonary function for patients with CHD. There was no significant publication bias in this study, and the results were highly reliable, but the included studies were heterogeneous with low methodological quality. Further high-quality and large-sample RCTs are suggested to be carried out to verify the reliability and extrapolation of the results.

## Figures and Tables

**Table 1:** Characteristics of 12 included studies in this meta-analysis.

Study	Type of patients	Follow-up, months	Rehabilitation nursing	Rehabilitation exercise program	*n*, m/f	Age, years	Course of CHD, years	NYHA
Chen et al.	CHD with PCI	6	Baduanjin	30 min/day, 5 days/week	43, 29/14	60.0 ± 10.9	New-onset	43 I-III
			Control	Maintain original habit of exercise	39, 30/9	61.5 ± 11.5	New-onset	39 I-III

Kang et al.	Stable CHD with CHF	6	Baduanjin + AWT	30 min/day, 5 days/week; AWT as control group	38, 21/17	68.4 ± 5.0	NR	21 II/17 III
			AWT	30–45 min/day, 5 days/week	38, 23/15	69.3 ± 5.3	NR	19 II/19 III

Liang	CHD	1	Baduanjin	30 min/day, 3–4 days/week	40, 22/18	56.4 ± 4.5	4.5 ± 2.3	40 I-II
			Control	Maintain original habit of exercise	40, 23/17	56.6 ± 4.3	4.3 ± 1.8	40 I-II

Liao	CHD with PCI	1	Baduanjin	30 min/day, 3 days/week	20, 12/8	54.6 ± 5.5	6.1 ± 2.4	NR
			Control	Maintain original habit of exercise	20, 11/9	54.8 ± 5.7	5.9 ± 2.3	NR

Ou	CHD with PCI	3	Baduanjin	30–60 min/time, 2 times/day	40, 21/19	52.2 ± 3.4	NR	NR
			Control	Maintain original habit of exercise	40, 22/18	52.0 ± 3.4	NR	NR

Shi et al.	CHD with PCI	3	Baduanjin + ER *∗*	15–30 min/day, 2–3 days/week; ER as control group	60, 47/13	58.3 ± 5.8	5.80 ± 2.61	60 I-III
			ER *∗*	60 min/day, 2–3 days/week	60, 52/8	58.9 ± 6.5	6.10 ± 2.96	60 I-III

Tang	CHD with PCI	3	Baduanjin	30 min/day, 5 days/week	46, 37/9	60.0 ± 8.7	NR	46 I-II
			Control	Maintain original habit of exercise	47, 38/9	61.4 ± 9.2	NR	47 I-II

Wang et al.	CHD	3	Baduanjin	40 min/day, 5 days/week	32, 34/30#	56.8 ± 12.3	NR	NR
			Control	Maintain original habit of exercise	32	56.8 ± 12.3	NR	NR

Wang et al.	Stable CHD with PCI or CABG	1	Baduanjin	30 min/day, 5–7 days/week	29, 21/8	64.3 ± 5.6	5.49 ± 5.15	3 I/24 II/2 III
			Control	Maintain original habit of exercise	29, 16/13	64.4 ± 6.6	6.11 ± 4.65	5 I/19 II/5 III

Xin	CHD	3	Baduanjin + AT	30 min/day, 3 days/week; AT as control group	44, 17/27	58.3 ± 7.1	4.37 ± 0.88	44 II
			AT	60 min/day, 3 days/week	44, 16/28	57.2 ± 7.2	4.32 ± 0.87	44 II

Xiong et al.	CHD with CHF	3	Baduanjin	30 min/day, 5–7 days/week	33, 20/13	70.3 ± 6.4	NR	33 II-III
			Control	Maintain original habit of exercise	30, 18/12	69.7 ± 7.2	NR	30 II-III

Zhang et al.	CHD with PTCA	0.5	Baduanjin	30 min/day, 7 days/week	43, 28/15	57.4 ± 13.1	New-onset	NR
			Control	Maintain original habit of exercise	41, 30/11	55.0 ± 16.5	New-onset	NR

AT, aerobic training; AWT, aerobic walking training; CABG, coronary artery bypass grafting; CHD, coronary heart disease, CHF, chronic heart failure; ER, Exercise rehabilitation (*∗*, means low intensity aerobic combined resistance exercise); F, female; *M*, male; NR, not reported; NYHA, The New York Heart Association; PCI, percutaneous coronary intervention; PTCA, percutaneous transluminal coronary angioplasty. #, sample size of male/female patients of total participants.

**Table 2:** Subgroup analyses results of LVEF.

Outcomes	No. of study	WMD (95% CI)	*P* value	Heterogeneity test	
				*I* ^2^ (%)	P_H_
Follow-up, months					
<1 month	2	5.09 (−0.40, 10.58)	0.07	57	0.13
3 months	4	4.18 (2.10, 6.26)	<0.0001	77	0.004
6 months	2	2.70 (0.38, 5.02)	0.02	0	0.34

Rehabilitation scheme					
Baduanjin vs. control	6	4.13 (2.12, 6.24)	<0.0001	70	0.005
Baduanjin + AT vs. AT	2	3.21 (1.24, 5.18)	0.001	0	0.82

New-onset					
Yes	2	1.94 (−0.94, 4.82)	0.19	0	0.54
No	6	4.32 (2.62, 6.03)	<0.00001	67	0.010

AT, aerobic training; CI, confidence interval; WMD, weighted mean difference.

## Data Availability

The authors confirm that the data supporting the findings of this study are available within the article and its supplementary materials.

## Additional Points

(1) Baduanjin can improve the levels of rehabilitation indicators in CHD patients. (2) Baduanjin is beneficial to cardiopulmonary function recovery in patients with CHD. (3) Subgroup analysis suggested a significant recovery effect of Baduanjin on LVEF. (4) This meta-analysis has no significant publication bias, and the results were highly reliable.

## Supplementary Materials

Supplementary Materials Figure S1: evaluating the methodological quality of included studies. Figure S2: subgroup analyses on LVEF, in termsoffollow-up time, rehabilitation scheme, and new-onset status. Table S1: retrieval strategies and results in PubMed. Table S2: retrieval strategies and results in Embase. Table S3: retrieval strategies and results in the cochrane library.

## References

[1] Tian Y., Deng P., Li B. (2019). Treatment models of cardiac rehabilitation in patients with coronary heart disease and related factors affecting patient compliance. *Reviews in Cardiovascular Medicine*.

[2] Deckers K., Schievink S. H. J., Rodriquez M. M. F. (2017). Coronary heart disease and risk for cognitive impairment or dementia: systematic review and meta-analysis. *PLoS One*.

[3] Anderson L., Brown J. P., Clark A. M. (2017). patient education in the management of coronary heart disease. *Cochrane Database of Systematic Reviews*.

[4] McMahon S. R., Ades P. A., Thompson P. D. (2017). The role of cardiac rehabilitation in patients with heart disease. *Trends in Cardiovascular Medicine*.

[5] Akyuz A. (2020). Exercise and coronary heart disease. *Advances in Experimental Medicine & Biology*.

[6] Anderson L., Thompson D. R., Oldridge N. (2016). Exercise-based cardiac rehabilitation for coronary heart disease. *Cochrane Database of Systematic Reviews*.

[7] Bai M. F., Wang X. (2020). Risk factors associated with coronary heart disease in women: a systematic review. *Herz*.

[8] Nichols S., McGregor G., Breckon J., Ingle L. (2021). Current insights into exercise-based cardiac rehabilitation in patients with coronary heart disease and chronic heart failure. *International Journal of Sports Medicine*.

[9] Guan Y., Hao Y., Guan Y., Wang H. (2020). Effects of Baduanjin exercise on essential hypertension: a meta-analysis of randomized controlled trials. *Medicine (Baltimore)*.

[10] Xiong X., Wang P., Li S., Zhang Y., Li X. (2015). Effect of Baduanjin exercise for hypertension: a systematic review and meta-analysis of randomized controlled trials. *Maturitas*.

[11] Chen X., Marrone G., Olson T. P. (2020). Intensity level and cardiorespiratory responses to Baduanjin exercise in patients with chronic heart failure. *ESC Heart Failure*.

[12] Zou L., SasaKi J. E., Wang H., Xiao Z., Fang Q., Zhang M. (2017). A systematic review and meta-analysis Baduanjin qigong for health benefits: randomized controlled trials. *Evidence-Based Complementary and Alternative Medicine*.

[13] Xiao X., Wang J., Gu Y., Cai Y., Ma L. (2018). Effect of community based practice of baduanjin on self-efficacy of adults with cardiovascular diseases. *PLoS One*.

[14] (2021). Analysis of the effect of baduanjin combined with aerobic endurance training on elderly patients with stable coronary heart disease complicated with chronic heart failure. *Chinese Journal of the Frontiers of Medical Science (Electronic Version)*.

[15] Chen M. G., Liang X., Kong L. (2020). Effect Of baduanjin sequential therapy on the quality of life and cardiac function in patients with ami after PCI: a randomized controlled trial. *Evidence-Based Complementary and Alternative Medicine*.

[16] (2020). Clinical observation on the effect of baduanjin on life quality of patients with coronary heart disease after stenting. *Bao Jian Wen Hui*.

[17] Liberati A., Altman D. G., Tetzlaff J. (2009). The PRISMA statement for reporting systematic reviews and meta-analyses of studies that evaluate health care interventions: explanation and elaboration. *PLoS Medicine*.

[18] Higgins J. P., Green S. (2008). *Cochrane Handbook for Systematic Reviews of Interventions*.

[19] Higgins J. P. T., Thompson S. G., Decks J. J., Altman D. G. (2003). Measuring inconsistency in meta-analyses. *British Medical Journal*.

[20] Higgins J. P. T., Thompson S. G. (2002). Quantifying heterogeneity in a meta-analysis. *Statistics in Medicine*.

[21] Egger M., Smith G. D., Schneider M., Minder C. (1997). Bias in meta-analysis detected by a simple, graphical test. *BMJ Clinical Research*.

[22] (2021). Effects of baduanjin on cardiopulmonary function in patients with coronary heart disease. *Chinese Health Care*.

[23] (2020). Clinical study of xinmaikang tablet combined with baduanjin on postoperative rehabilitation of coronary heart disease with PCI. *Journal of North Pharmacy*.

[24] (2021). Clinical effect of baduanjin in cardiac rehabilitation after percutaneous coronary intervention. *Chinese Journal of Rehabilitation Medicine*.

[25] (2019). Effects of baduanjin exercise on exercise tolerance of elderly patients after percutaneous coronary intervention. *Nursing of Integrated Traditional Chinese and Western Medicine*.

[26] Wang H. (2018). Effect of baduanjin on cardiopulmonary function in patients with coronary heart disease during cardiac rehabilitation. *Journal of Hunan University of Chinese Medicine*.

[27] (2021). The effect of baduanjin on exercise cardiopulmonary function and quality of life in patients with stable coronary artery disease: a randomized controlled trial. *Journal of Traditional Chinese Medicine*.

[28] (2018). Effects of Baduanjin on cardiopulmonary function in patients with coronary heart disease during cardiac rehabilitation. *Health Care Today*.

[29] (2016). Effect of Baduanjin on chronic heart failure patients with coronary heart disease. *Modern Medicine Journal of China*.

[30] (2021). Study on the effect of Baduanjin exercise on the stage I rehabilitation of acute myocardial infarction after PTCA. *Modern Journal of Integrated Traditional Chinese and Western Medicine*.

[31] Lavie C. J., Milani R. V. (2011). Cardiac rehabilitation and exercise training in secondary coronary heart disease prevention. *Progress in Cardiovascular Diseases*.

[32] Kavanagh T., Mertens D. J., Hamm L. F. (2003). Peak oxygen intake and cardiac mortality in women referred for cardiac rehabilitation. *Journal of the American College of Cardiology*.

[33] Vanhees L., Fagard R., Thijs L., Amery A. (1995). Prognostic value of training-induced change in peak exercise capacity in patients with myocardial infarcts and patients with coronary bypass surgery. *The American Journal of Cardiology*.

[34] Keteyian S. J., Brawner C. A., Savage P. D. (2008). Peak aerobic capacity predicts prognosis in patients with coronary heart disease. *American Heart Journal*.

[35] Kodama S., Saito K., Tanaka S. (2009). Cardiorespiratory fitness as a quantitative predictor of all-cause mortality and cardiovascular events in healthy men and women: a meta-analysis. *JAMA*.

[36] Wang X. Q., Pi Y. L., Chen P. J. (2016). Traditional Chinese exercise for cardiovascular diseases: systematic review and meta-analysis of randomized controlled trials. *Journal of American Heart Association*.

[37] Zhang H., Chang R. (2019). Effects of exercise after percutaneous coronary intervention on cardiac function and cardiovascular adverse events in patients with coronary heart disease: systematic review and meta-analysis. *Journal of Sports Science and Medicine*.

[38] Kao W., Jessup M. (1994). Exercise testing and exercise training in patients with congestive heart failure. *The Journal of Heart and Lung Transplantation*.

[39] Yu M., Li S., Li S., Li J., Xu H., Chen K. (2018). Baduanjin exercise for patients with ischemic heart failure on phase-II cardiac rehabilitation (BEAR trial): study protocol for a prospective randomized controlled trial. *Trials*.

[40] Ziaeian B., Fonarow G. C. (2016). Epidemiology and aetiology of heart failure. *Nature Reviews Cardiology*.

