# Supplementary material for: Updated Meta-Analysis Assessing Effects of Baduanjin on Cardiopulmonary Functions of Patients with Coronary Heart Disease
Source: Evid Based Complement Alternat Med. 2022 Sep 29;2022:3913082. doi: 10.1155/2022/3913082 (PMC9536900; doi:10.1155/2022/3913082)
Supplement: Supplementary Materials — Figure S1: evaluating the methodological quality of included studies. Figure S2: subgroup analyses on LVEF, in termsoffollow-up time, rehabilitation scheme, and new-onset status. Table S1: retrieval strategies and results in PubMed. Table S2: retrieval strategies and results in Embase. Table S3: retrieval strategies and results in the cochrane library. [file 3913082.f1.docx]

The retrieval time: 20220809

**Table S1 Retrieval strategies and results in PubMed**

| Search | Query | Items found |
| --- | --- | --- |
| #1 | "coronary disease"[MeSH Terms] OR "coronary heart disease"[All Fields] | 256962 |
| #2 | "ischemic heart disease"[All Fields] OR "Ischemic cardiomyopathy"[All Fields] OR "myocardial infarction"[All Fields] OR "acute coronary syndrome"[All Fields] OR ("angina pectoris"[MeSH Terms] OR "angina pectoris"[All Fields] OR "stenocardia"[All Fields]) OR "coronary artery disease"[All Fields] | 439201 |
| #3 | #1 OR #2 | 554842 |
| #4 | "Baduanjin"[All Fields] | 168 |
| #5 | #3 AND #4 | 10 |

**Table S2 Retrieval strategies and results in Embase**

| Search | Query | Items found |
| --- | --- | --- |
| #1 | coronary heart disease.mp. or exp ischemic heart disease/ | 754612 |
| #2 | coronary disease.mp. or exp coronary artery disease/ | 377259 |
| #3 | stenocardia.mp. or exp angina pectoris/ | 104565 |
| #4 | ("Ischemic cardiomyopathy" or "myocardial infarction" or "acute coronary syndrome").mp | 381748 |
| #5 | #1 OR #2 OR #3 OR #4 | 931903 |
| #6 | Baduanjin.mp | 204 |
| #7 | #5 AND #6 | 10 |

**Table S3 Retrieval strategies and results in the cochrane library**

| Search | Query | Items found |
| --- | --- | --- |
| #1 | MeSH descriptor: [Coronary Disease] explode all trees | 14704 |
| #2 | (“coronary heart disease” OR “ischemic heart disease” OR “Ischemic cardiomyopathy” OR “myocardial infarction” OR “acute coronary syndrome” OR stenocardia OR “coronary artery disease”):ti,ab,kw (Word variations have been searched) | 59721 |
| #3 | #1 OR #2 | 62305 |
| #4 | (Baduanjin):ti,ab,kw (Word variations have been searched) | 170 |
| #5 | #3 AND #4 | 12 |


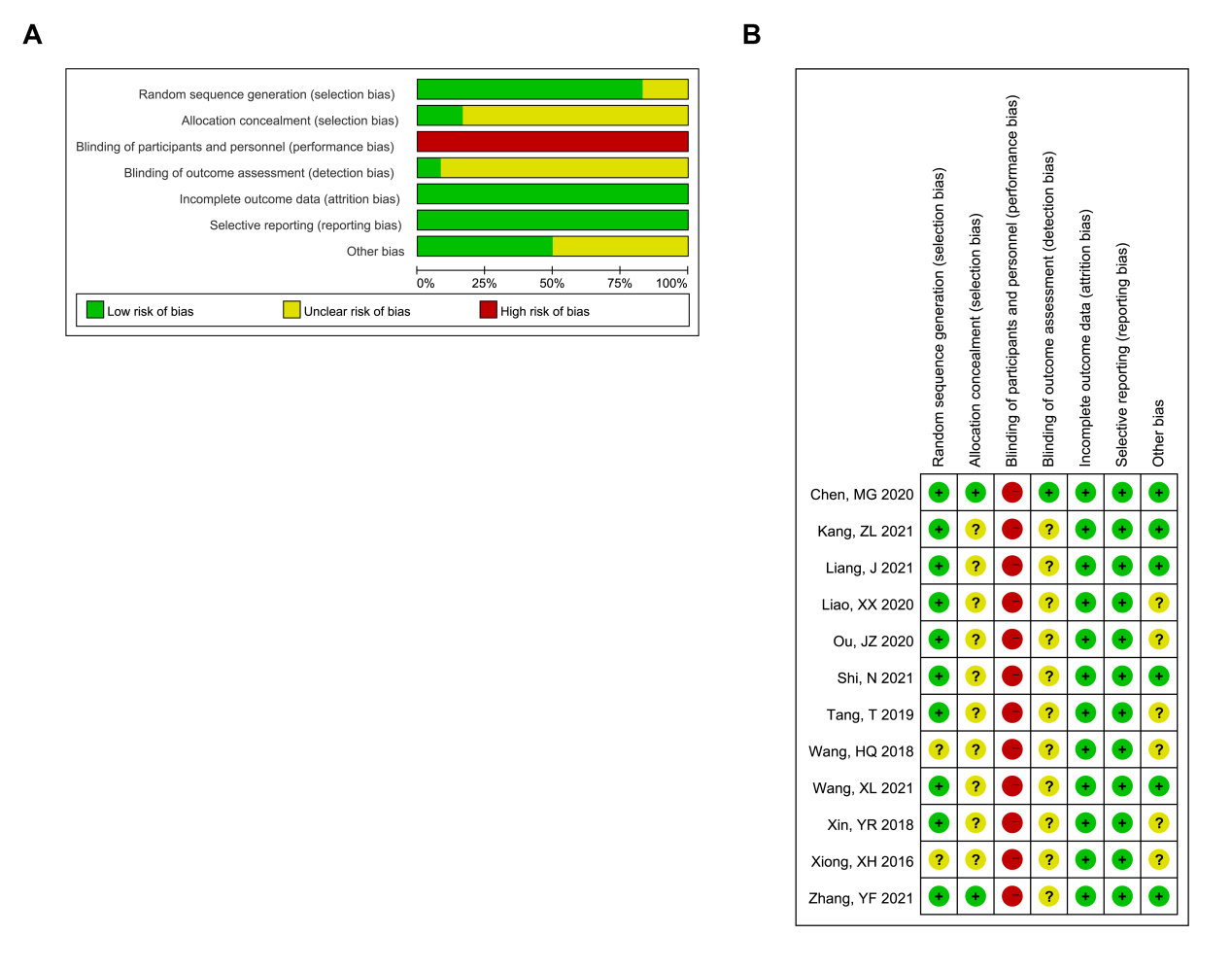


Figure S1

**
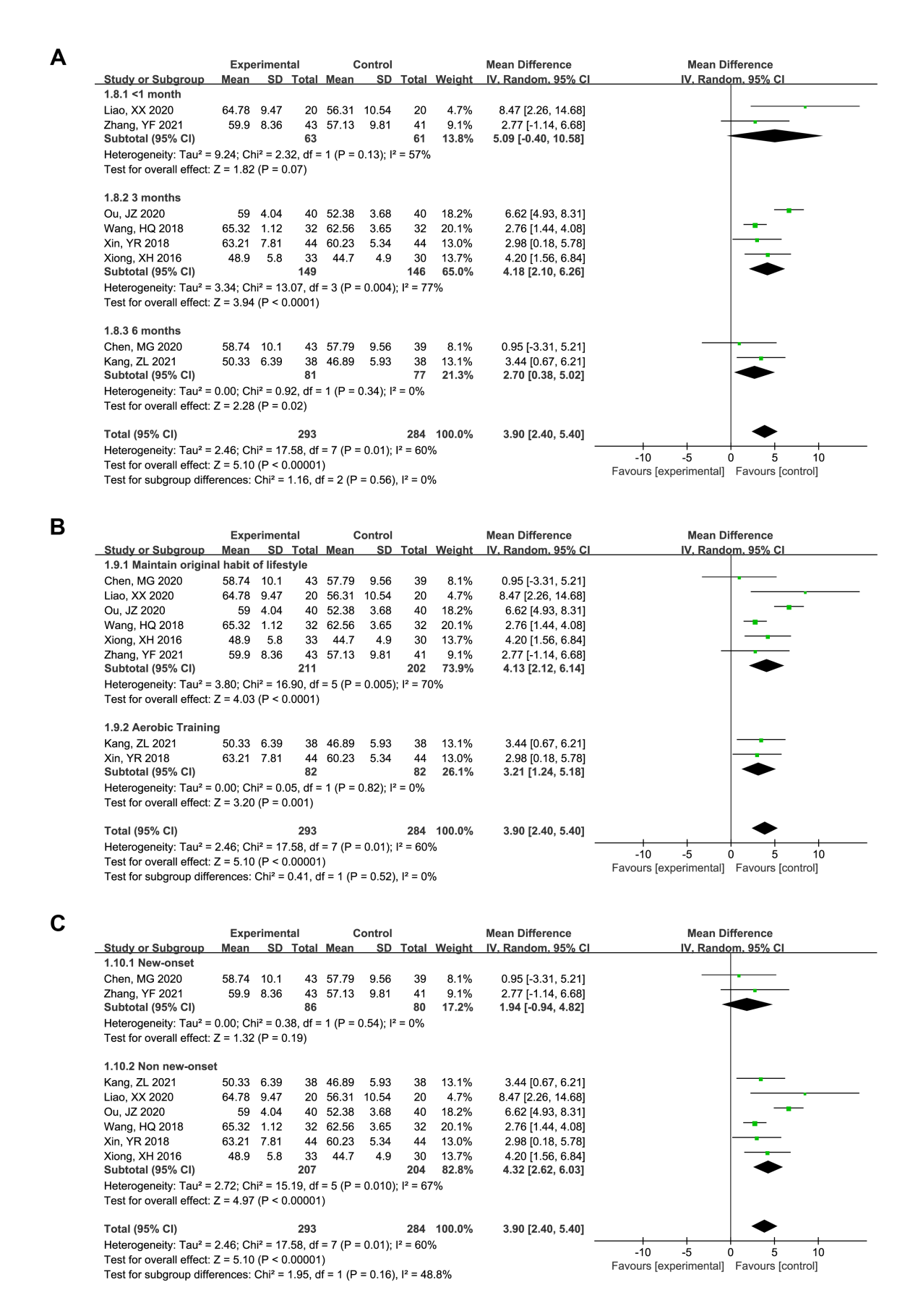
**

Figure S2
